# Comprehensive analysis of genomic alterations and novel prognostic biomarkers, and establishment of prediction models of metastasis in metastatic non-small cell lung cancer

**DOI:** 10.7150/jca.97070

**Published:** 2025-01-01

**Authors:** Kangwei Wang, Meifeng Ye, Zexun Mo, Xiaomei Huang, Yujun Li, Shuquan Wei

**Affiliations:** 1 Department of Pathology, Guangzhou Red Cross Hospital, Guangzhou Red Cross Hospital of Jinan University, Guangzhou, China.; 2 Department of Pathology, Guangzhou First People's Hospital, South China University of Technology, Guangzhou, China.; 3 Department of Pulmonary and Critical Care Medicine, Guangzhou First People's Hospital, South China University of Technology, Guangzhou, China.

**Keywords:** Non-small cell lung cancer, Next-generation sequencing, Prediction model, Metastasis, Survival analysis.

## Abstract

**Introduction:** Most patients with non-small cell lung cancer (NSCLC) have metastases at initial diagnosis. However, the comprehensive molecular characteristics and factors associated with its metastases are still needed.

**Methods:** Tumor sequencing of 556 cancer-related genes was performed on 114 Chinese NSCLC patients. A distinct genomic profile was identified in metastatic patients compared to those without metastases. Kaplan-Meier method was used to analyze the associations between clinical outcomes, clinical characteristics, and mutated genes. The Fisher test and Lasso logistic regression analysis were employed to identify factors related to metastasis and to develop prediction models.

**Results:** Male, squamous cell lung carcinoma, and smokers showed strikingly higher TMB levels in all NSCLCs. The metastatic group had a significantly higher proportion of patients aged ≥ 70 years and in stage III-IV. *TP53* was the most frequent mutation in both groups, and *EGFR* tended to be higher in the metastatic group. The copy number variation events occurred more frequently in the metastatic group. Additionally, predictive models for metastasis (AUC = 0.828), pleural metastasis (AUC = 0.582), and multisite metastasis (AUC = 0.559) were established. Females, and *EGFR* +, *ASXL2-*, and *STK11-* cases had better overall survival (OS). Lung adenocarcinoma, and *KMT2D-* and *STK11-* cases had better progression-free survival (PFS). NSCLC metastasis was associated with poor OS and poor PFS.

**Conclusions:** Our study provided a comprehensive analysis of genomic alterations in metastatic NSCLCs, identified novel prognostic biomarkers, and provided three predictive models for metastasis, which may have potential implications for personalized treatment strategies.

## Introduction

Lung cancer is a major contributor to global fatalities related to cancer, primarily owing to its high metastatic potential 1-3. About 85% of lung cancers are non-small-cell lung cancer (NSCLC) including lung adenocarcinoma (LUAD), squamous cell lung carcinoma (SCLC), and other histological subtypes. Unfortunately, NSCLC has a five-year survival rate of less than 18% 2, 4. While surgical resection proves highly successful in the initial phases, a considerable number of patients are unfortunately diagnosed with distant metastases, which makes them ineligible for surgical intervention 5. In addition, approximately 65% of NSCLCs experience recurrence and metastasis after surgery with unsatisfactory treatment 6. Enhancing the timely detection of metastases can extend patients' survival and enhance their quality of life 7. Understanding the molecular characteristics of and correlated risk factors of metastatic lung cancer has important clinical significance.

Prior research has indicated that lung cancer metastasis is linked to several clinical factors, including age, gender, primary tumor locations, smoking history, and pathology 8-10. However, the molecular mechanisms of lung cancer metastasis remain largely unknown. Next-generation sequencing (NGS) technology has revolutionized the field of cancer research by enabling the comprehensive analysis of the cancer genome. Several gene mutations related to metastasis have been identified in some studies including a small number of genes 11-13. Therefore, there is still a need for a broader panel that includes genes of significant importance for the accurate identification and effective management of NSCLC.

There, we aim to elucidate the molecular characteristics of lung cancer metastasis by a panel of 556 genes associated with cancer. Additionally, we developed three predictive models for lung cancer metastasis by using these molecular features and correlated risk factors, which could potentially aid in the identification of NSCLC patients who are at a heightened risk of developing metastasis, allowing for the implementation of personalized treatment strategies and surveillance measures.

## Materials and Methods

### Patient collection

We enrolled patients from Guangzhou First People's Hospital and collected clinical data from the medical records. Inclusion criteria: 1) Patients must meet the minimum age requirement of 18 years. 2) Patients with a confirmed pathological diagnosis of NSCLC, which must be made by two experienced pathologists and confirmed by the appropriate diagnostic procedures. 3) All patients must be presented with an initial diagnosis of lung cancer and have not undergone any prior anti-tumor treatment, such as chemotherapy, radiation therapy, targeted therapy, or immunotherapy. 4) Patients who had undergone surgical resection or puncture for the diagnosis or treatment of their condition and had sufficient qualified NGS sequencing data. 5) Patients who had complete clinical and pathological data available for analysis. Exclusion criteria: 1) Patients with a history of other concurrent diseases or conditions that could significantly affect the expression profile of genomic alterations or novel prognostic biomarkers, including autoimmune diseases, severe infections or others. 2) Patients who had received any form of chemotherapy, radiation therapy, or targeted therapy prior to surgery or puncture. 3) Patients with incomplete or missing clinical and pathological data. Only patients meeting all the inclusion criteria and none of the exclusion criteria were included in the final study cohort. To ensure a robust study population, we initially gathered a total of 134 patients who had undergone surgical resection or puncture. However, 20 patients were excluded from the study due to the insufficient tissue sample for sequencing analysis, lack of complete clinical information or with primary tumors other than lung cancer. The study received approval from the ethics committee of Guangzhou First People's Hospital (K-2024-002-01). The privacy of all patients' personal information was strictly maintained, and every patient provided their informed consent by signing a consent form.

### DNA extraction and sequencing

Experienced pathologists conducted a thorough macroscopic examination of the specimens to determine their clinicopathological subtypes. For all patients, blood samples were used as the corresponding normal control. The QIAamp DNA FFPE tissue kit (Qiagen) was employed to extract genomic DNA from Formalin-fixed, paraffin-embedded (FFPE) tissues, and quantified by Qubit 4.0 using the dsDNA HS Assay Kit (ThermoFisher Scientific). Additionally, matched white blood cell DNA was extracted using the DNeasy Blood & Tissue kit (Qiagen). Libraries were prepared from extracted DNA and sequenced as paired-end reads on Illumina HiSeq6000 using PE150 sequencing chemistry (Illumina). Library construction sample requirements: sample quality: gDNA, no serious degradation by agarose gel electrophoresis; FFPE DNA, fragment length > 500 bp; total sample size: 50 ~ 500 ng; sample purity: OD260/OD280 = 1.8 ~ 2.0; OD260/OD230 = 2.0 to 2.5. The libraries were subjected to enrichment using a panel specifically designed to target 556 cancer-related genes, which was developed by Shanghai Tongshu Biotechnology Co., Ltd. The average depth of sequencing required for tissues is ≥ 1000×. Libraries were quantified by qPCR using KAPA Library Quantification kit (KAPA Biosystems). Library fragment size was determined by Bioanalyzer 2100 (Agilent Technologies). Reads were filtered based on both high mapping quality and base quality scores (≥30), with mutant reads requiring support from both positive and negative strands. The variant allele frequency (VAF) is ≥ 1%. The Burrows-Wheeler Aligner (BWA) software was utilized to align the clean paired-end reads to the human genome build hg19 (UCSC). For alignment optimization, variant calling, and annotation, we utilized GATK 14, MuTect 14, and VarScan 15, respectively.

### Mutational signatures

We utilized non-negative matrix factorization from the R package NMF to analyze the mutational signatures. The base substitutions were categorized into 6 directions (C > T, C > A, C > G, T > C, T > G, and T > A). By applying the NMF algorithm, we deconstructed the mutational signatures. Cosine similarity was employed as a metric to assess the resemblance between our signatures and those in COSMIC.

### Copy number variation (CNV)

We detect the shared CNV area in all samples by GISTIC 2.0. The parameters of the GISTIC 2.0 method were set as follows: a significance threshold of Q ≤ 0.05 was used to assess the significance of the change. A confidence level of 0.95 was utilized when identifying the peak interval. These values were derived from analyzing the distribution of log2 ratios to detect peaks linked to copy number states. The area greater than the length of the chromosome arm was 0.98 as the standard of the chromosome arm level. The analysis of somatic CNV was conducted using the facet software.

### Statistical analysis

Categorical associations were identified by using Fisher's exact test. The Mann-Whitney U test was used to analyze variations in continuous variables among the groups. Progression-free survival (PFS) and overall survival (OS) were assessed using the Kaplan-Meier method along with the log-rank test. Statistical significance was determined at a two-sided p-value of less than 0.05.

## Results

### Patient characteristics

In our study, a cohort of 114 NSCLC patients was examined. The median age of the patients was 67 years (44 to 90 years). Among them, 58.8% (67/114) were under the age of 70, and 66.7% (76/114) were male. Smoking history was present in 54.4% (62/114) of the patients, and 11.4% (13/114) had a history of drinking. The majority of patients (71.9%, 82/114) had metastasis. Histopathological examination revealed 76.3% (87/114) of LUADs, 14.0% (16/114) of SCLC, and 9.7% (11/114) of other types of NSCLC. Furthermore, out of 114 patients, 101 of them which is 88.6% had tumors in stage III or IV. Among the patients, 73.7% exhibited Eastern Cooperative Oncology Group (ECOG) scores of 0-1. **Supplementary Figure 1** shows the range of ECOG scores observed and the correlations with stage and metastasis. Patients with stage Ⅳ had a wide distribution of ECOG scores, with the highest proportion in all three groups of ECOG, albeit without significant differences (*p* = 0.35, **Supplementary Figure 1A**). With the increase of ECOG score, the proportion of patients with stage Ⅳ gradually increased. Similarly, patients with metastasis had a broad distribution of ECOG scores and were the highest in all three groups of ECOG, without significant differences (*p* = 0.20, **Supplementary Figure 1B**). A summary of the basic patient information is in **Table 1**.

### Mutation landscape of patients with NSCLC

NGS data revealed a total of 1,251 somatic mutations from 112 patients. The mutational landscape of NSCLC is summarized in **Supplementary Figure 2A**. Notably, the most prominent and significant variations were mutations in *TP53* (72/112, 64.29%) and *EGFR* (46/112, 41.07%), followed by mutations in *LRP1B* (32/112, 28.57%), *KRAS* (19/112, 16.96%), *ATM* (14/112, 12.50%), *FAT1* (14/112, 12.50%), *PTPRD* (12/112, 10.71%), and *SPTA1* (12/112, 10.71%). Other frequently mutated genes included *KEAP1* (11/112, 9.82%) and *KMT2C* (10/112, 8.93%). Missense mutations were the predominant mutation type identified, with C > A being the most frequently occurring base mutation (**Supplementary Figure 2A**). Moreover, we investigated the association between the top 30 genes and clinical characteristics. Females, smokers, and LUADs were more likely to have *EGFR* mutations (all *p* < 0.005). Whereas, *KRAS* mutations were more commonly detected in smokers and drinkers (both *p* < 0.05).

Additionally, the mutational signatures we extracted and 30 known COSMIC signatures are shown in **Supplementary Figure 2B**. The analysis of genetic characteristics revealed extensive heterogeneity among the patients, with most of them exhibiting signatures 1, 2, 4, 13, 15, 18, 20, 22, and 24, which are linked to age, smoking, viral infection, and other characteristics.

Somatic copy number alterations (SCNAs) in NSCLC were analyzed in all tumor tissue samples. A total of 20 significant copy number gain peaks were identified, including 8q24.21 (*MYC*), and 39 significant copy number loss peaks, including 12q13.12 (*KMT2D*), 2q22.1 (*LRP1B*), 13q14.2 (*BRCA2*, *RB1*), and 12q24.31 (*POLE*) (**Supplementary Figure 2C**).

To explore the biological functions of mutated genes, enrichment analyses were performed using the Kyoto Encyclopedia of Genes and Genomes (KEGG) and Gene Ontology (GO) databases in NSCLC. **Supplementary Figure 2D** shows the commonly altered signaling pathways enriched by KEGG enrichment analyses. The result showed that genes showed significant enrichment in both the PI3K-Akt signaling pathway and pathways related to EGFR kinase inhibitor resistance. The GO enrichment analyses revealed that the mutant genes exhibited significant enrichment primarily in terms related to the chromosomal region, transferase complexes, positive regulation of kinase activity, and DNA-binding transcription factor binding (**Supplementary Figure 2E**). The results of GO and KEGG analyses demonstrated that the high-frequency mutations were notably concentrated in the PI3K-Akt signaling pathway and pathways associated with EGFR kinase inhibitor resistance, which are closely implicated in tumor development.

**Figure 1:**
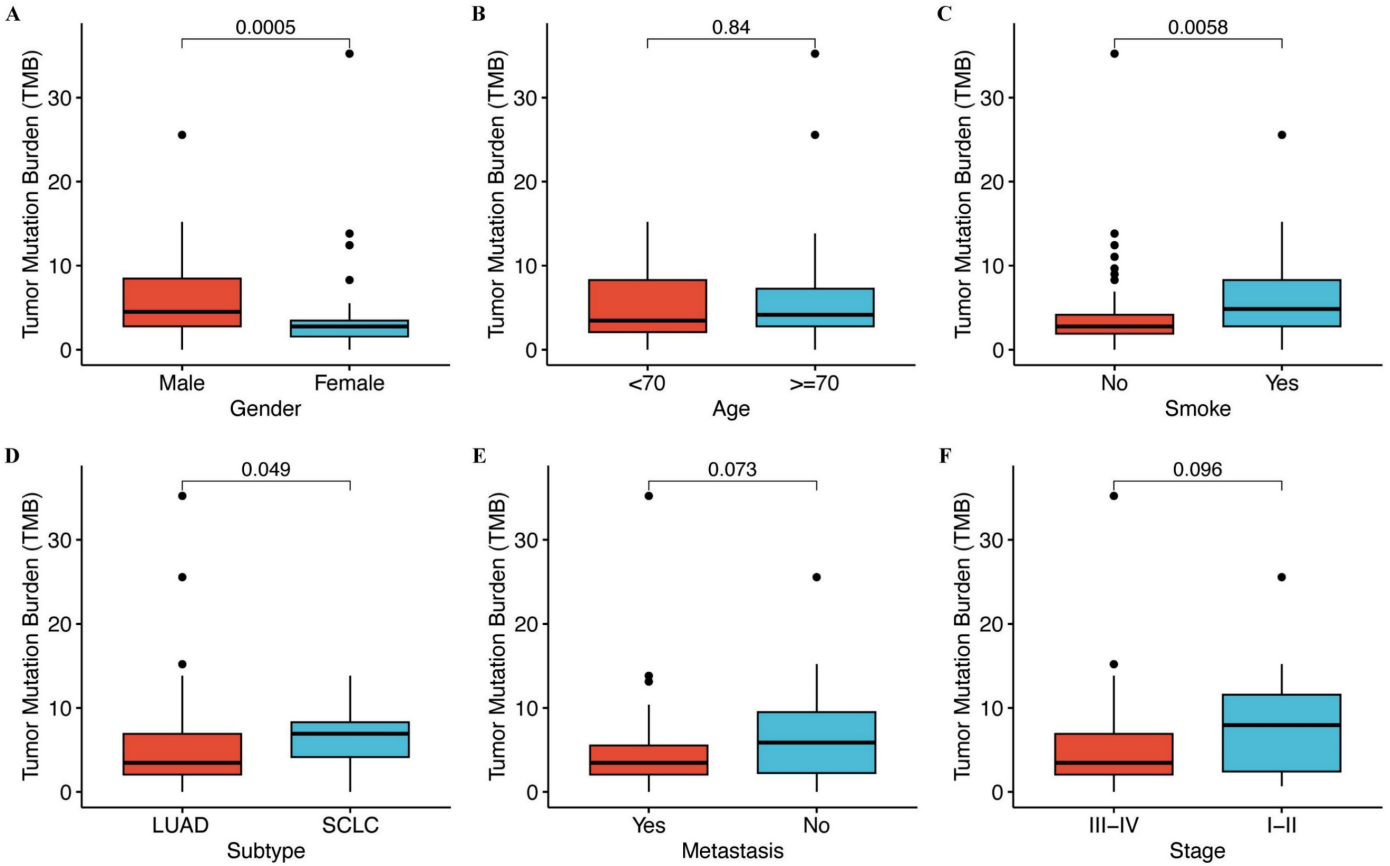
**Correlation between TMB and clinical features.** (**A**) The level of TMB is compared between male and female patients. (**B**) The level of TMB is compared between patients with age < 70 years and ≥ 70 years. (**C**) The level of TMB is compared between smokers and non-smokers. (**D**) The level of TMB is compared between lung adenocarcinoma (LUAD) and squamous cell lung carcinoma (SCLC). (**E**) The level of TMB is compared between metastatic and non-metastatic patients. (**F**) The level of TMB is compared between different stages.

We analyzed to assess the correlation between tumor mutation burden (TMB) and clinical characteristics. Our findings revealed no significant association between TMB and age, metastasis, or stage (**Figure 1**), whereas TMB tended to be positively correlated with gender (*p* = 0.0005), histological type (*p* = 0.049), and smoking status (*p* = 0.0058). Males, SCLC, and smokers showed strikingly high TMB levels (**Figure 1**).

### Clinical characteristics and mutational profile of patients with or without metastasis

To explore the factors contributing to metastasis development in NSCLC, we categorized the participants into two groups depending on whether they had metastasis or not. The group of patients with metastasis had a notably higher proportion of individuals aged 70 years or older (*p* < 0.05, **Table 2**) and in advanced stages III/IV (*p* < 0.01, **Table 2**) compared to the non-metastatic group.

**Figure 2:**
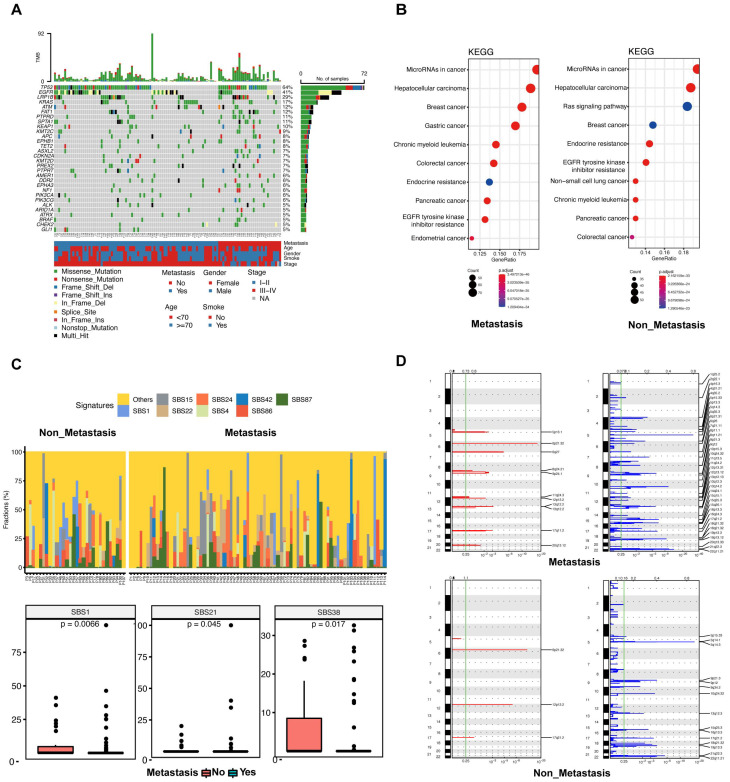
** Landscapes of patients with or without metastasis.** (**A**) The most frequently mutated genes in patients with and without metastasis. (**B**) Kyoto Encyclopedia of Genes and Genomes (KEGG) enrichment for metastatic patients (left) and non-metastatic patients (right). (**C**) The top 8 mutational signatures in metastatic and non-metastatic patients. (**D**) The focal copy number variations in metastatic (upper) and non-metastatic (lower) patients by GISTIC 2.0 analysis. Chromosome positions are indicated along the y-axis. On the x-axis, focal deletions or amplifications are depicted with horizontal blue or red bars, respectively. The green line represents the significance threshold of q < 0.25 (the false discovery rate after multiple hypothesis testing).

The top 30 mutated genes in the metastatic and non-metastatic groups were demonstrated in **Figure 2A**. In the metastatic group, the *TP53* gene showed mutations in 48 out of 82 cases (58.54%), followed by the *EGFR* gene with mutations in 36 out of 82 cases (43.90%), and the *LRP1B* gene with mutations in 20 out of 82 cases (24.39%), whereas the most frequent mutation in the non-metastatic cases was *TP53* (24/32, 75%), flowed by *LRP1B* (12/32, 37.5%) and *EGFR* (10/32, 31.25%). In comparison with metastatic group, the mutations of* FAT1* (33.33%), *ASXL2* (20.83%), *HIST1H3B* (20.83%),* EPHA3* (20.83%),* MST1R* (12.50%),* BRAF* (16.67%),* ARID1A* (16.67%) and *TSHR* (16.67%) were significantly more frequent in non-metastatic group (all *p* < 0.05).

The KEGG enrichment result revealed that the predicted functions were significantly enriched in the tumor microRNA signaling pathway and hepatocellular carcinoma pathway in patients with or without metastasis (**Figure 2B**). The Ras signaling pathway was found to be significantly enriched in the non-metastatic group (**Figure 2B**). Functional enrichment of the two groups was similar according to GO analysis. (**Supplementary Figure 3A, B**).

Tumors in both groups were more prone to C > A mutations as well as C > T mutations, and there was no statistically significant variation observed between the two groups across these six categories of base substitution (**Supplementary Figure 3C**). Different types of cancer exhibit unique mutational signatures, indicating the involvement of various mutational processes. These signatures are reflective of the diverse genetic backgrounds and exposure etiologies associated with each cancer type. We calculated the contribution of signatures of mutational processes in NSCLC (**Figure 2C upper**). The mutational signature analysis showed that the top 8 signatures consisted of signature 15, signature 24, signature 42, signature 87, signature 1, signature 22, signature 4, and signature 86 (**Figure 2C lower**). Signature 1 (a cell division/mitotic clock) and signature 38 (indirect effect of ultraviolet light) were enriched in the non-metastatic group (**Figure 2C lower**). Conversely, signature 21 (associated with defective DNA mismatch repair) was found to be more prevalent in the metastatic group (**Figure 2C lower**). Taken together, these data suggest that mutational processes were not the same between patients with or without metastasis.

Chromosomal instability correlates with tumor metastasis and is a driver of metastasis. We conducted a CNV analysis on the primary genome data of two groups; and explored whether the difference in genome structure caused the occurrence of metastasis. The result showed that the copy number deletion was significantly more than the copy number amplification in each group (**Figure 2D**). In both amplification and deletion, the metastatic group exhibited a significantly higher level of copy number variation compared to the non-metastatic group (**Figure 2D**). In the metastatic group, we identified eleven prominent peaks showing increased copy numbers, such as 8q24.21 (*MYC*), and thirty-nine significant peaks displaying decreased copy numbers, including 19p13.3 (*STK11*), 13q14.2 (*BRCA2*, *RB1*), 12q13.12 (*KMT2D*), 2q22.1 (*LRP1B*), and 18q21.32 (*NARS*) (**Figure 2D**). In the non-metastatic group, there were 3 significant peaks of copy number gain, including 12p13.2 (*ETV6*), and 15 significant peaks of copy number loss, including 19p13.3 (*STK11*), 17q21.2 (*BRCA1*), and 5p15.33 (*TERT*) (**Figure 2D**). Collectively, these results suggest that CNVs could be significant factors in the advancement of disease and the spread of tumors in lung cancer.

### Prediction models for metastases of NSCLC

To explore variables associated with metastases of NSCLC, we conducted Fisher's exact test on clinical information and the mutated genes in different groups. The significantly associated (*p* < 0.05) genes or clinical information obtained were selected as candidate features. These candidate features were then used to build the model using the Lasso regression method to predict metastasis in patients.

**Figure 3:**
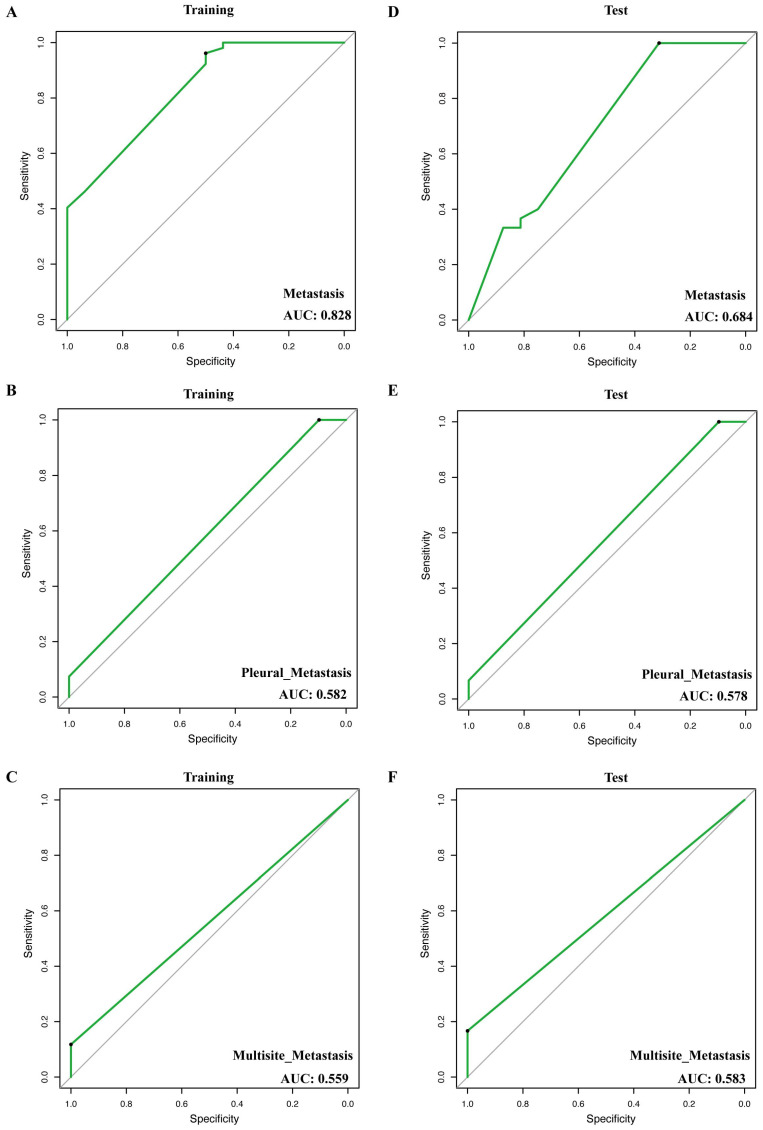
**Receiver operator characteristic (ROC) curve of models for prediction of metastasis, pleural metastasis, and multisite metastasis.** (**A**, **D**) ROCs of model of training and test groups for prediction of metastasis. (**B**, **E**) ROCs of model of training and test groups for prediction of pleural metastasis. (**C**, **F**) ROC of model of training and test groups for prediction of multisite metastasis.

In total, we construct 3 models for the prediction of metastasis, including metastasis (any organ of metastasis), pleural metastasis, and multisite metastasis (more than one organ of metastasis). In the training cohort, the AUC (area under the curve) of the metastasis prediction model (*EPHA3* + *FAT* + *ASXL2* + *HIST1H3B* + *TSHR* + *MST1R*) was 0.828 (**Figure 3A**), of pleural metastasis (*NF1*+ *FGF3*) was 0.582 (**Figure 3B**) and of multisite metastasis (*FANCC* + *ERBB4*) was 0.559 (**Figure 3C**). Notably, in the test cohort, the 3 models also showed stable performance (AUC = 0.684, 0.578, and 0.583, respectively, **Figure 3D-F**).

### Prognostic impact of clinical and genomic characteristics

**Figure 4:**
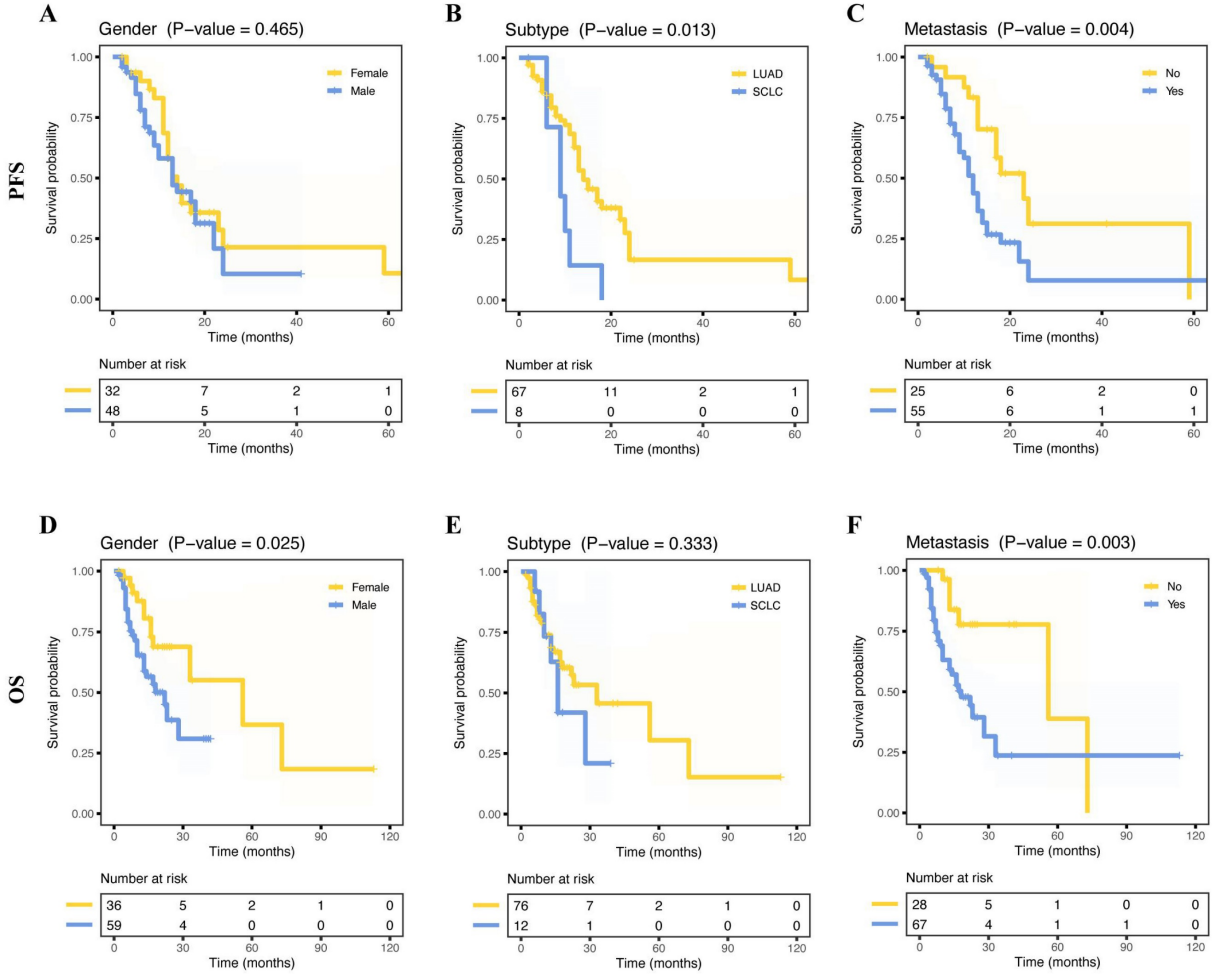
** Survival curves for progress-free survival (PFS) and overall survival (OS) for patients based on different clinical information.** Kaplan-Meier survival curves of PFS for patients, including gender (**A**), histology subtype (**B**), and metastasis status (**C**). Kaplan-Meier survival curves of OS for patients, including gender (**D**), histology subtype (**E**), and metastasis status (**F**).

The median OS of 95 patients was 15 months (range, 2 - 113 months) and the median PFS of 80 patients was 12 months (range, 2 - 69 months). Fifteen months was the median OS for 95 patients, with a range of 2 to 113 months, while 12 months was the median PFS for 80 patients, with a range of 2 to 69 months. Out of the 114 patients examined, relapse occurred in 82 cases, accounting for 71.9%. LUAD had a better PFS than SCLC (**Figure 4B,** *p* = 0.013), and female patients had a better OS than male patients (**Figure 4D,** *p* = 0.025). As expected, NSCLC metastasis had a poorer PFS (**Figure 4C**, *p* = 0.004) and poorer OS (**Figure 4F**, *p* = 0.003). However, age, smoking status, drinking status, and stage (I/II vs. III/IV) did not show any significant prognostic differences (all *p* > 0.05, **Supplementary Figure 4A-D**, **I-L**). Furthermore, we further explored the correlation between metastatic organs and prognosis. The result showed that pleural, bone, or multisite metastasis was not significantly associated with OS and PFS (**Supplementary Figure 4E-G, M-O**).

**Figure 5:**
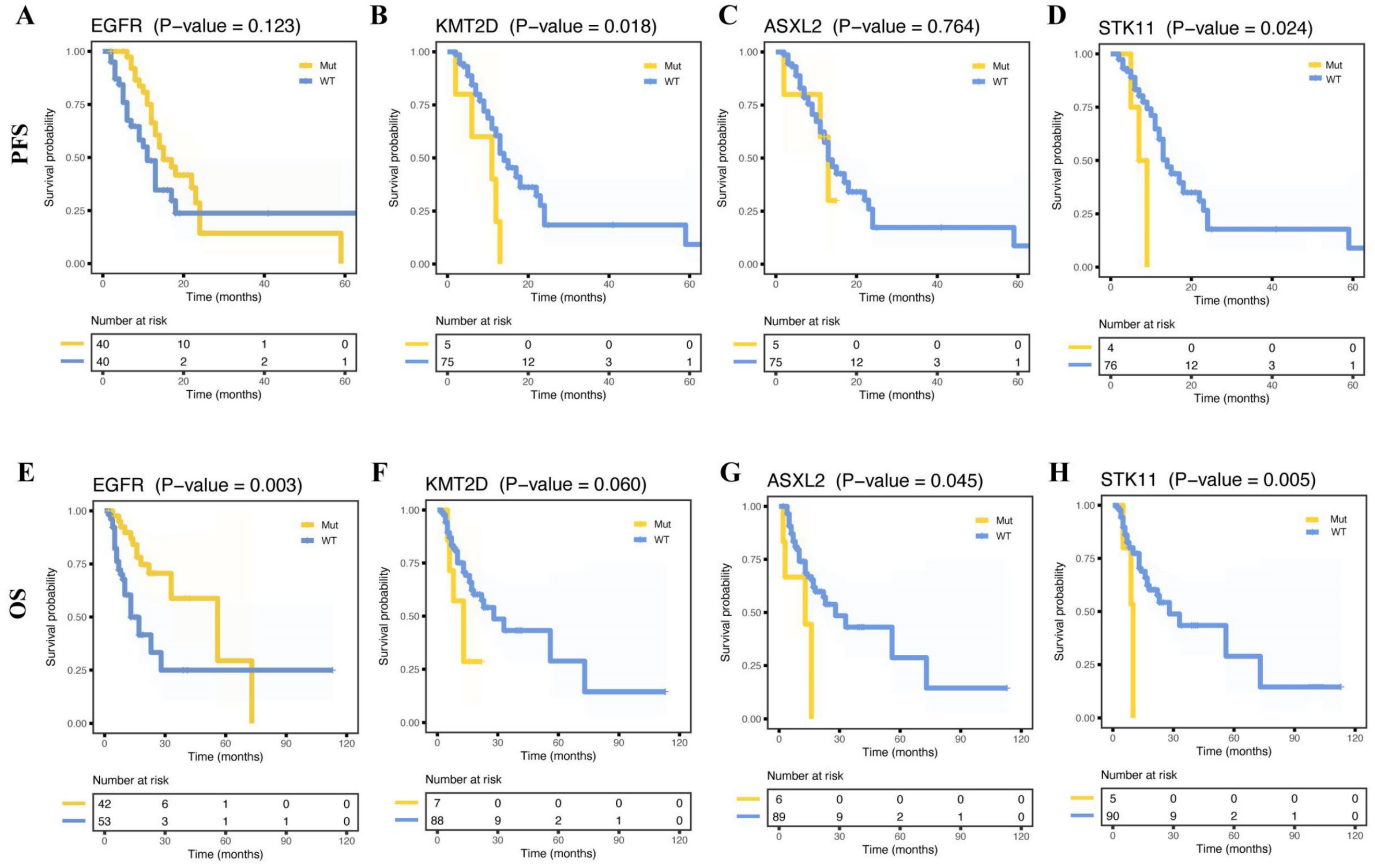
** Survival curves for progress-free survival (PFS) and overall survival (OS) for patients with and without certain mutations.** Kaplan-Meier survival curves of PFS for patients with and without mutant *EGFR* (**A**), *KMT2D* (**B**), *ASXL2* (**C**), and *STK11* (**D**). Kaplan-Meier survival curves of OS for patients with and without mutant *EGFR* (**E**), *KMT2D* (**F**), *ASXL2* (**G**), and *STK11* (**H**).

The study compared the relationship between long-term prognosis and metastasis-associated genes based on mutation status. We found that a similar PFS (**Figure 5A**, *p* = 0.123) was observed in two groups and a longer OS in patients with *EGFR* mutations (**Figure 5E**, *p* = 0.003). Whereas cases with *KMT2D* or *STK11* mutations showed poorer PFS than those without (**Figure 5B**, **D**, *p* = 0.018, *p* = 0.024, respectively). And *ASXL2* or *STK11* wild-type cases had better OS (**Figure 5G-H**, *p* = 0.045, *p* = 0.005, respectively). However, OS and PFS did not differ from the other mutation-positive and -negative groups. Nevertheless, there were no differences in OS and PFS between the mutation-positive and -negative groups. Furthermore, no significant prognostic differences in TMB were observed between the two groups (**Supplementary Figure 4H, P**).

Finally, an examination of the relationship between the treatment and patient outcomes revealed interesting findings. Specifically, individuals receiving a chemotherapy combination with other therapies tend to have extended PFS and overall survival OS compared to those undergoing chemotherapy only (**Supplementary Figure 5A-B**). Similarly, patients treated with targeted therapy combined with chemotherapy tended to have better survival than those treated with targeted therapy alone (**Supplementary Figure 5C-D**). However, patients subjected to a combination of chemotherapy and immunotherapy demonstrated similar outcome in comparison to their counterparts receiving chemotherapy (**Supplementary Figure 5E-F**). These results underscore the importance of tailoring treatment strategies to individual patient needs and highlight the potential benefits of personalized medicine in improving prognosis and outcomes for cancer patients.

## Discussion

NSCLC is a significant global contributor to cancer-related mortality, with a majority of patients already having metastases upon initial diagnosis. Furthermore, the 5-year survival rate for NSCLC is suboptimal 16. Although NSCLC gene sequencing has received more attention, the understanding of the genetic profile and underlying mechanisms contributing to the progression of metastatic cancer remains limited. In this research, we examined the genetic mutational characteristics associated with the metastasis of NSCLC from 114 Chinese patients by using targeted NGS and developed 3 metastasis prediction models. By analyzing the gene mutations in samples from metastatic patients and non-metastatic patients, we were able to identify potential genetic alterations that may contribute to the metastatic process.

Among NSCLCs, we observed a consistent pattern with previous studies, where *TP53*, *EGFR*, *LRP1B*, and *KRAS* were identified as the most frequently mutated genes 17, 18. The distribution of genetic alterations in Chinese and Caucasian patients differed. According to a study conducted on the American population, it was found that the gene with the highest frequency of mutations is *KRAS*, followed by *EGFR* 19. Some EGFR tyrosine kinase inhibitors (TKIs) are now available for treating advanced NSCLC with common *EGFR*-sensitizing mutations 20. The presence of *KRAS* mutations in resected lung cancer is associated with a poor prognosis. However, determining the mutant status of both *KRAS* and *EGFR* can significantly enhance treatment decisions, especially when considering the use of kinase inhibitors 21, 22. Mutations in the *LRP1B* gene have been documented to correlate with a high tumor mutation burden, and they have also been linked to a more favorable response to immunotherapy 18. The mutated genes in NSCLC could potentially be targeted, offering a wider range of possibilities and strategies for treating NSCLC.

In addition, we analyzed the genetic mutations in non-metastatic and metastatic groups and suggested that these mutations could play crucial roles in tumor metastasis. The most frequent mutations in both groups were *EGFR* and *TP53*. Additionally, we observed a higher expression of *EGFR* in the non-metastatic group compared to the metastatic group (43.90% vs. 31.25%), and *STK11* and *RET* are only expressed in the metastatic group. These findings suggest that these mutations could potentially play a key role in the promotion of lung cancer metastasis. *EGFR* mutation has been reported to be a relapse-related factor in LUAD 23 and may be positively correlated with lung metastasis of NSCLC 24. Approximately 50% of human cancers exhibit p53 loss or mutation, and mutant p53 not only lacks tumor suppressor activity but also promotes malignant progression 25, 26. *TP53* mutations were predominantly early and persistent, appearing before metastatic spread, and associated with a heightened likelihood of metastasis 26-28. The cause of this effect may be linked to chromosomal instability or drug resistance, but more research is necessary 29.

Our findings have shown a notable variation in CNV between the two groups. According to certain studies, the amplification of *MYC* may be responsible for promoting metastases in cases of lung cancer 30, 31. By presenting proof of the functional significance of aneuploidy in facilitating metastasis, it was demonstrated that *MYC* amplification enhances metastatic processes by attracting a larger number of tumor-associated macrophages, thereby promoting increased invasion into the bloodstream 32. Intriguingly, in addition to *MYC* amplification, we identified multiple distinct CNVs that varied between the two groups, including arm 20q gain and arm 2q loss, which may contribute to the metastasis of lung cancer. These findings suggest that CNVs could have a significant impact on the advancement of the disease and the spread of tumors in lung cancer.

Furthermore, we used the mutant genes that significantly correlated with the organ tropism metastases of NSCLC to construct metastasis prediction models. By combining these factors, we were able to develop three robust models for the prediction of metastasis, pleural metastasis, and multisite metastasis, respectively. The AUC of the metastasis prediction model (AUC = 0.828) is greater than 0.6 which accurately predicted the likelihood of metastasis in lung cancer patients. The predictive model showed robust performance in distinguishing between metastatic and non-metastatic patients. This indicates that the genetic mutations can provide valuable insights into the metastatic potential of lung cancer. The features exhibited by the models suggest that performing a comprehensive assessment of gene mutations and other risk factors would be extremely advantageous in predicting metastases even organ-specific metastases in NSCLC. Besides, it is recommended to incorporate the examination of pertinent organs in the post-treatment monitoring of patients with these characteristics.

Gender as a prognostic factor for lung cancer continues to be a contentious issue. Some studies did not observe any notable disparity in prognosis between male and female patients 33, 34. However, a larger study reported a negative correlation between male and lung cancer prognosis 35. Similarly, our findings revealed a significant link between gender and OS (p = 0.025), while no significant relationship was observed with PFS (p = 0.465) in our study. In line with earlier discoveries, our results suggested that LUAD has a better prognosis than SCLC 36. To definitively determine the prognostic role of clinical factors, larger sample sizes with more detailed stratification would be necessary.

Currently, the primary clinical approach for treating lung cancer involves a comprehensive treatment strategy centered around chemotherapy. However, the efficacy of chemotherapy in advanced NSCLC patients is generally low, with success rates typically ranging from 20% to 40% 6, 37. In recent years, molecular targeted therapy has emerged as a promising new approach to treating cancer. This treatment method involves the use of drugs that target specific sites within cancer cells, such as EGFR-TKI, thereby inhibiting their growth with precision 38. Some studies found that the disease control rate of targeted therapy combined with chemotherapy could be effectively improved and prolong the PFS of the patients 39, 40. Our study showed similar results without significant differences (**Supplementary Figure 5C, 5D**). The breakthrough discovery of immune checkpoint inhibitors (ICIs) has significantly changed the landscape of cancer immunotherapy 41, achieving remarkable success in treating a variety of advanced cancers 42, 43. Notably, first-line ICI combined chemotherapy has emerged as a cutting-edge approach for treating stage IV NSCLC without target gene mutations and with PD-L1 expression 44. Previous studies have shown that chemotherapy combined with immunotherapy significantly improves response rates and longer PFS or OS compared to chemotherapy alone 40, 45, 46. However, there was no difference in our results, which may be due to the small sample size (**Supplementary Figure 5E-F**). Additionally, our study findings suggest that compared with chemotherapy-only, chemotherapy combined with other treatments has a better prognosis for survival (**Supplementary Figure 5A-B**). Thereby, the effect of treatment on prognosis may influence the effect of clinical and genomic characteristics on prognosis to some extent, and further comprehensive research is necessary with a larger sample size.

It should be noted that this study has certain limitations. Firstly, the research was conducted at a single institution with a relatively small sample size, which could not avoid possible confounding factors and selective bias, potentially restricting the applicability of our results. Subsequent investigations involving larger cohorts are warranted to confirm and validate our findings. Secondly, our analysis focused on using unpaired patient samples to identify the genetic mutations in the non-metastatic and metastatic groups, which may not more precise exploration of underlying molecular mechanisms and evolutionary patterns. Furthermore, integrating other omics data could offer a more holistic understanding of the molecular alterations associated with lung cancer metastasis.

## Conclusions

Our research emphasizes the role of genetic mutations in promoting the spread of lung cancer. Some specific mutations allowed us to develop three predictive models for metastasis, which may have implications for personalized treatment strategies. More studies are warranted to confirm our findings and explore additional genetic alterations associated with lung cancer metastasis.

## Supplementary Material

Supplementary figures.

## Figures and Tables

**Table 1:** Clinical characteristics of patients with NSCLC included in this study.

Characteristics	N (%)
**Total**	114 (100)
**Age**	[Median 67; range 44 - 90]
< 70	67 (58.8)
≥ 70	47 (41.2)
**Gender**	
Male	76 (66.7)
Female	38 (33.3)
**Smoking**	
Never smoke	52 (45.6)
Current or former	62 (54.4)
**Drinking**	
Never drink	101 (88.6)
Current or former	13 (11.4)
**Metastasis**	
Yes	82 (71.9)
No	32 (28.1)
**Histology**	
Squamous	16 (14.0)
Adenocarcinoma	87 (76.3)
Other	11 (9.7)
**Stage at diagnosis**	
I-II	12 (10.5)
III-IV	101 (88.6)
Unknow	1 (0.9)
**ECGO**	
0-1	84 (73.7)
2-3	26 (22.8)
>3	4 (3.5)

SCLC: squamous cell lung carcinoma; LUAD: lung adenocarcinoma; ECGO: Eastern Cooperative Oncology Group.

**Table 2:** Correlation between clinical features and metastasis.

Characteristics	Metastasis (N = 82, %)	Non_Metastasis (N = 32, %)	Total (N = 114, %)	pvalue
Age				< 0.05
<70	43(52.44)	24(75.00)	67(58.77)	
≥ 70	39(47.56)	8(25.00)	47(41.23)	
Smoking				0.65
No	39(47.56)	13(40.63)	52(45.61)	
Yes	43(52.44)	19(59.38)	62(54.39)	
Drinking				1
No	73(89.02)	28(87.50)	101(88.60)	
Yes	9(10.98)	4(12.50)	13(11.40)	
Histology				0.95
LUAD	62(75.61)	25(78.13)	87(76.32)	
SCLC	12(14.63)	4(12.50)	16(14.04)	
Other	8(9.76)	3(9.38)	11(9.65)	
Stage				< 0.01
I/II	0(0.00)	12(37.50)	12(10.53)	
III/IV	82(100.00)	19(59.38)	101(88.60)	
Unknow	0(0.00)	1(3.13)	1(0.88)	

LUAD: lung adenocarcinoma; SCLC: squamous cell lung carcinoma.
