# Supplementary material for: Comprehensive analysis of genomic alterations and novel prognostic biomarkers, and establishment of prediction models of metastasis in metastatic non-small cell lung cancer
Source: J Cancer. 2025 Jan 1;16(1):339–50. doi: 10.7150/jca.97070 (PMC11660132; doi:10.7150/jca.97070)
Supplement: Supplementary file 1 — Supplementary figures. [file jcav16p0339s1.pdf]

Supplemental information for

Comprehensive analysis of genomic alterations and novel prognostic biomarkers, and establishment of prediction models of metastasis in metastatic non-small cell lung cancer

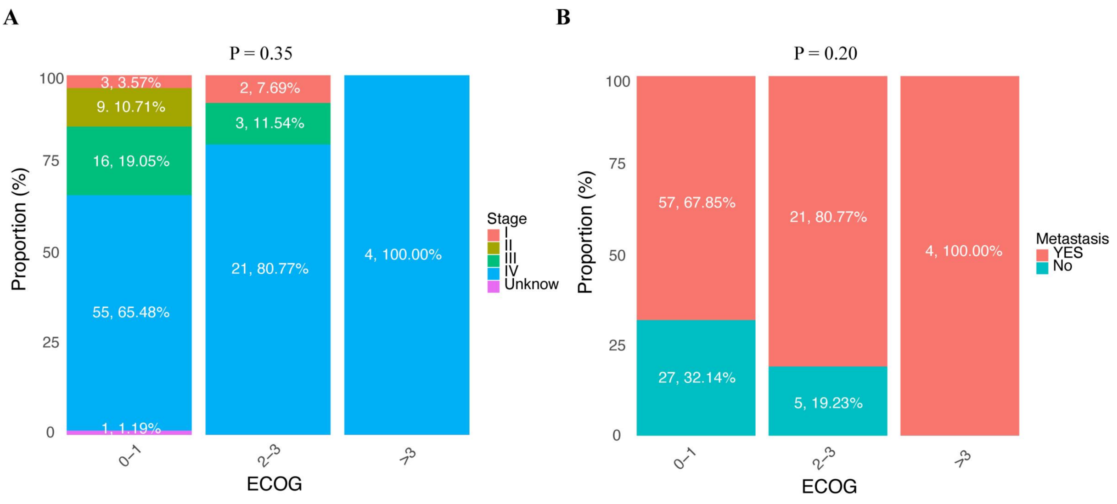

Figure S1 The range of ECOG scores and the correlations with stage (A) and metastasis (B).

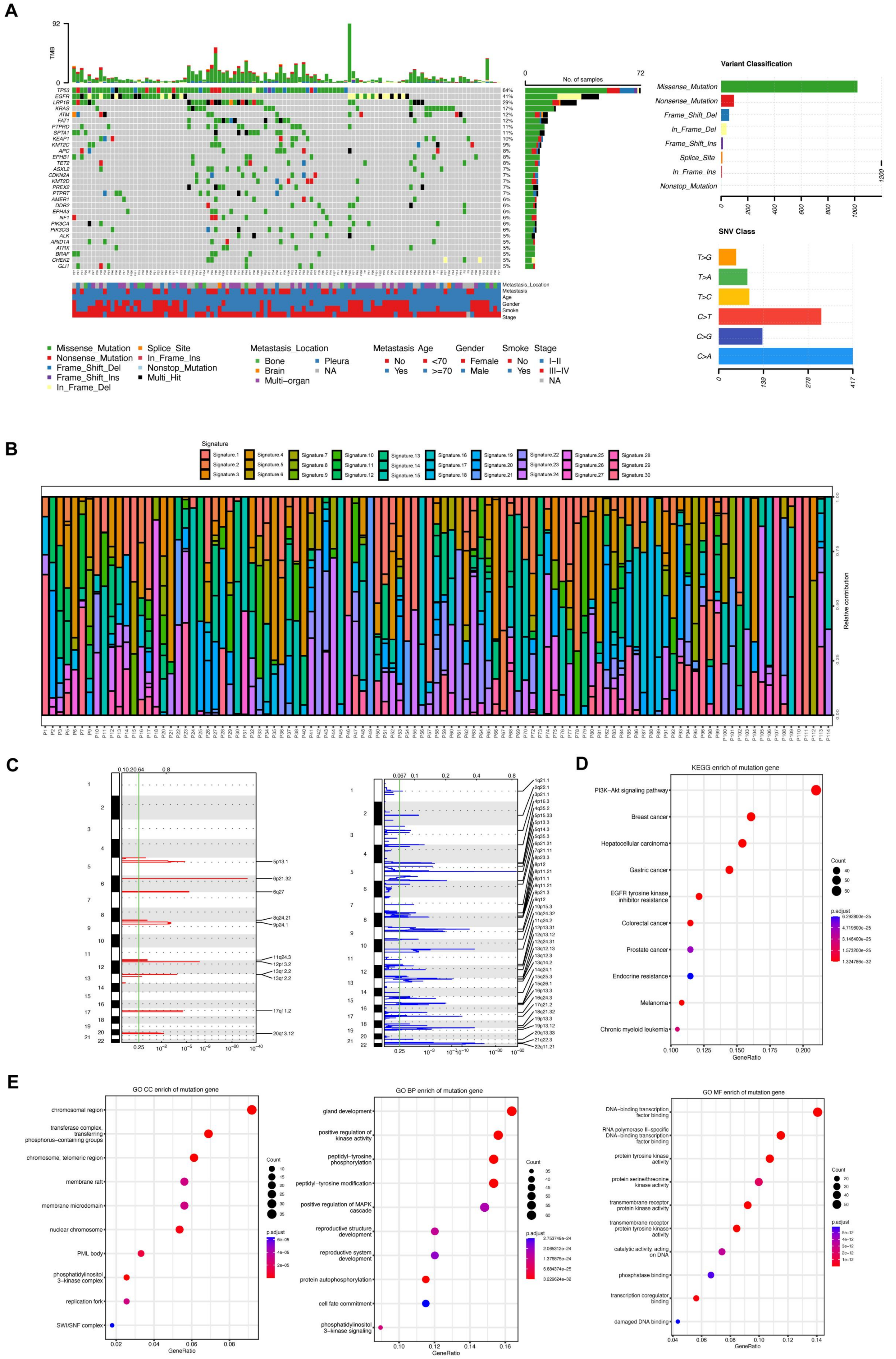

Figure S2 Mutational spectrums, signatures, and gene function analysis in NSCLCs. (**A**) Mutation spectrum of 112 samples of patients with NSCLC. (**B**) Mutational signatures in each patient. (**C**) The

focal copy number deletions in the NSCLCs by GISTIC 2.0 analysis. Chromosome positions are indicated along the y-axis. On the x-axis, focal deletions or amplifications are depicted with horizontal blue or red bars, respectively. The green line represents the significance threshold of $q < 0.25$ (the false discovery rate after multiple hypothesis testing). (**D**) Kyoto Encyclopedia of Genes and Genomes (KEGG) enrichment for patients with NSCLC. (**E**) Gene Ontology (GO) enrichment for patients with NSCLC, including cellular component (CC), biology process (BP), and molecular function (MF), respectively.

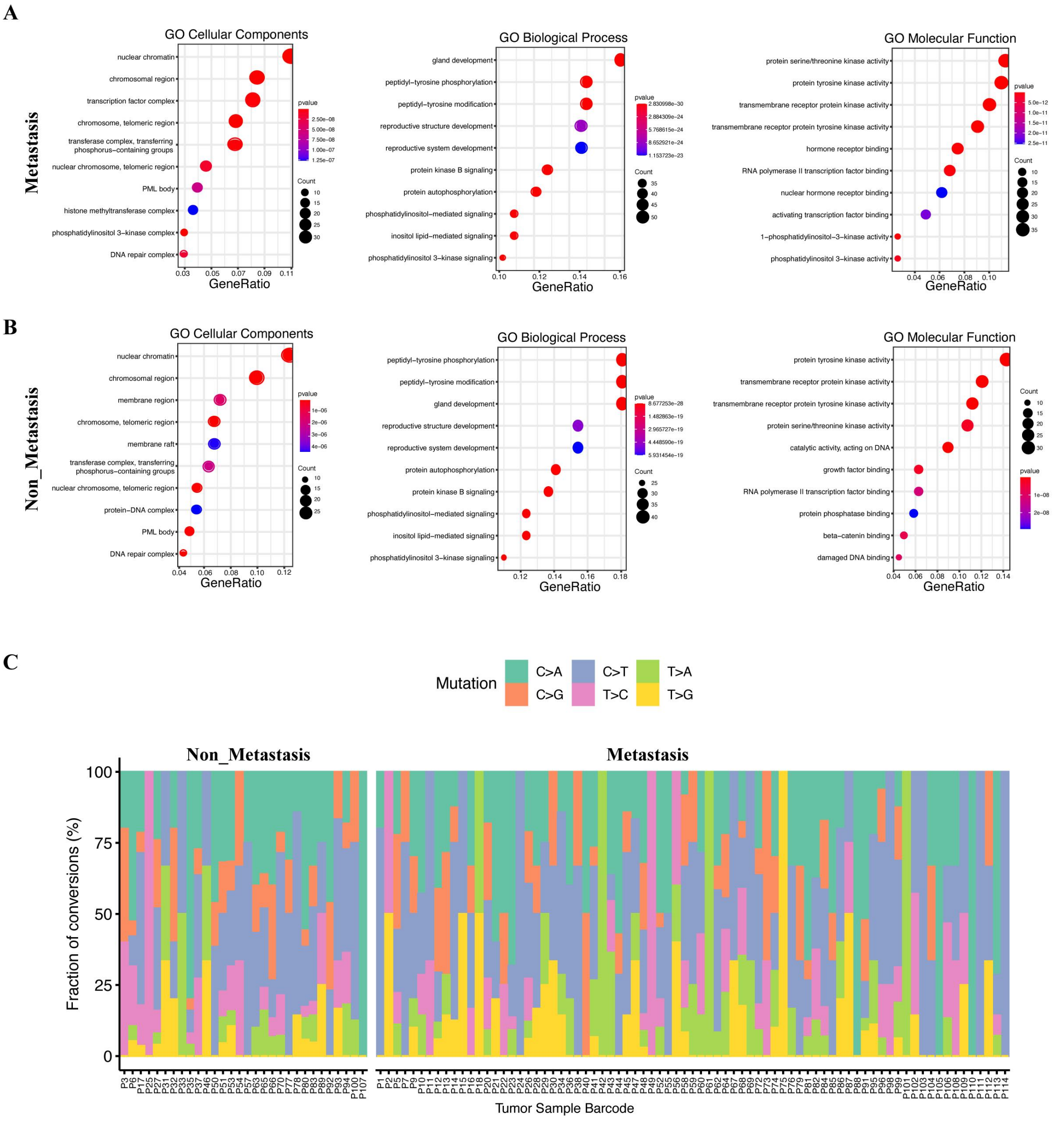

Figure S3 Gene Ontology (GO) enrichment and type of transition or transversion analysis for patients with or without metastasis. (**A**) GO enrichment analysis for metastatic patients, including cellular component (CC), biology process (BP), and molecular function (MF), respectively. (**B**) GO enrichment analysis for non-metastatic patients, including cellular component (CC), biology process (BP), and molecular function (MF), respectively. (**C**) The bar plot shows each type of transition or transversion in non-metastatic and metastatic patients.

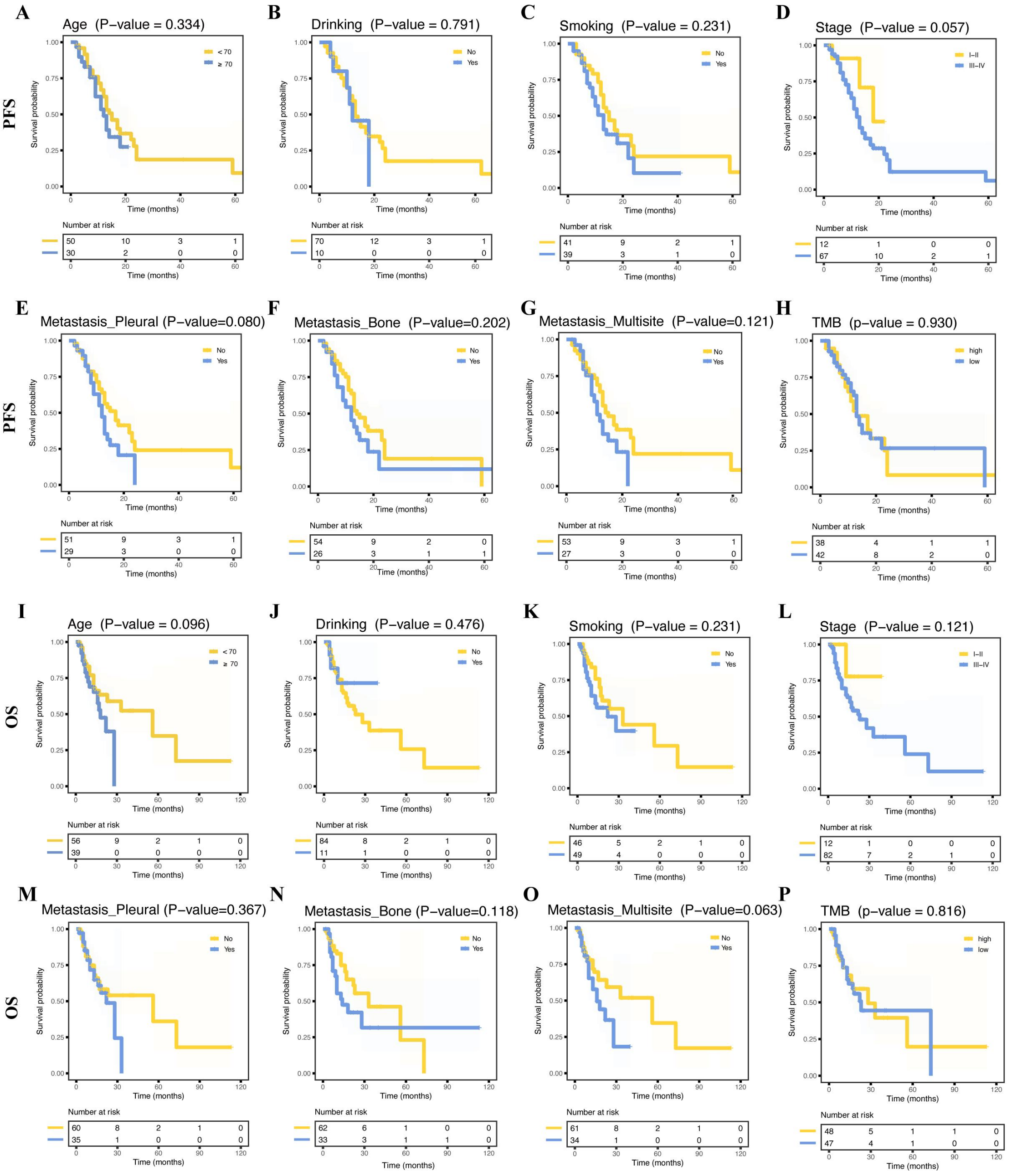

Figure S4 Survival curves for progress-free survival (PFS) and overall survival (OS) for patients based on different clinical information and TMB. Kaplan–Meier survival curves of PFS for patients, including age (**A**), drinking status (**B**), smoking status (**C**), stage (**D**), pleural metastasis (**E**), bone metastasis (**F**), multisite metastasis (**G**) and TMB level (**H**). Kaplan–Meier survival curves of OS for patients, including age (**I**), drinking status (**J**), smoking status (**K**), stage (**L**), pleural metastasis (**M**), bone metastasis (**N**), multisite metastasis (**O**) and TMB level (**P**).

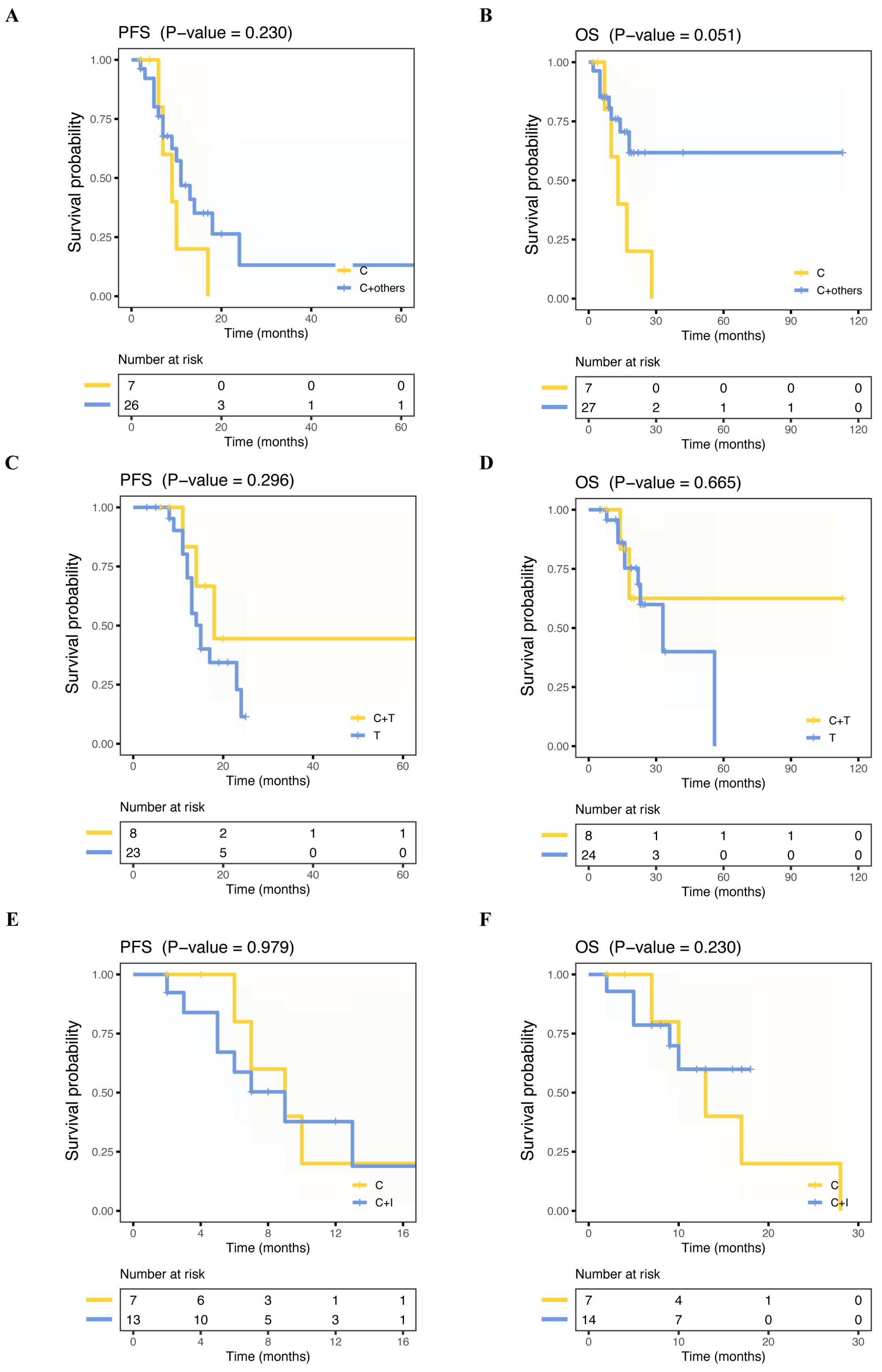

Figure S5 Survival curves for progress-free survival (PFS) and overall survival (OS) for patients

with different treatments. Kaplan-Meier curve of PFS (**A**) and OS (**B**) for the patients received chemotherapy and chemotherapy combine with other therapies, including antiangiogenic, immunotherapy or targeted therapy. Kaplan-Meier curve of PFS (**C**) and OS (**D**) for the patients received targeted therapy and targeted therapy combine with chemotherapy. Kaplan-Meier curve of PFS (**E**) and OS (**F**) for the patients received chemotherapy and chemotherapy combine with immunotherapy. C, chemotherapy; C+others, chemotherapy combine with antiangiogenic, immunotherapy or targeted therapy. I, immunotherapy; T, targeted therapy; PFS, progression-free survival; OS, overall survival.
